# Advances in Molecular Tools for Fungal Pathogen Detection and Identification in Common Bean: A Systematic Review

**DOI:** 10.1007/s12033-026-01572-3

**Published:** 2026-03-31

**Authors:** Khulasande Siyabonga Ngcobo, Sandiswa Figlan, Molemi Rauwane

**Affiliations:** 1 Department of Botany, Nelson Mandela University, P O Box 77000, Port Elizabeth, 6031 South Africa; 2 Department of Biotechnology and Food Technology, University of Johannesburg, Doornfontein, Johannesburg, 2094 South Africa; 3 Department of Agriculture and Animal Health, School of Agriculture and Life Sciences, College of Agriculture and Environmental Sciences, University of South Africa, Roodepoort, South Africa

**Keywords:** *Phaseolus vulgaris* L., Fungal disease detection, Metabarcoding, Next-generation sequencing

## Abstract

Common bean (*Phaseolus vulgaris* L.) is crucial for global food security but is highly vulnerable to fungal pathogens that impact yield and quality. Molecular tools have transformed pathogen detection, enabling accuracy, sensitivity, and rapid diagnostics. This review, following PRISMA 2020 guidelines, synthesizes recent advancements in molecular methods, including PCR, qPCR, isothermal amplification, next-generation sequencing (NGS), and bioinformatics pipelines for the detection and identification of fungal pathogens in common bean. While PCR-based methods dominate, NGS and metabarcoding offer deeper insights into fungal communities. Challenges remain, such as primer specificity, high costs, and the need for robust databases. Additionally, the growing use of NGS highlights the demand for trained bioinformaticians to manage large datasets. Addressing these challenges through training programs and interdisciplinary collaboration is crucial for improving pathogen detection. This review underscores the potential of emerging molecular technologies to enhance fungal disease management and calls for scalable, affordable solutions suited to diverse agricultural systems.

## Introduction

*Phaseolus vulgaris* L*.* (*P. vulgaris*) is a significant food legume within the Fabaceae family [15], also referred to as the common bean, string bean, field bean, French bean, and kidney bean, among other designations (Musango et al., 2016). It is considered one of the most nutritious crops, providing high protein, fiber, essential micronutrients, and complex carbohydrates (Hegay et al., 2013). It is cultivated worldwide for its edible leaves, green immature pods, and dried seeds (Gioia et al. 2019). However, global consumption (21 g/person/day) has remained stagnant over the past few decades [14], influenced not only by biotic stresses such as pathogens [11] but also by abiotic, socioeconomic, cultural, and market factors [3].

Fungal diseases represent a major biotic constraint to bean production, and their frequency and severity are increasing with climate change [8]. The most important fungal pathogens affecting common bean include *Alternaria* spp., *Aspergillus* spp., *Fusarium oxysporum*, *Botrytis* spp., *Colletotrichum lindemuthianum*, *Sclerotinia sclerotiorum*, *Rhizoctonia solani*, and *Cladosporium* spp., among others [9, 10]. These pathogens infect plant tissues such as leaves, seeds, pods, and roots, causing wilting, necrosis, anthracnose lesions, and seed rot [12]. Dissemination of the fungal spores occurs through infected seed, soil, wind, water, and human activity, making rapid detection essential for disease management.

Historically, the detection and identification of fungal pathogens have relied on morphology-based methods such as microscopy and culture-based techniques, which are labor-intensive, time-consuming, and prone to misidentification due to overlapping symptoms [19, 20]. While Yadav et al. [22] suggested that effective detection strategies were still limited at that time, molecular diagnostics were already being applied. Conventional PCR, serological assays, and biochemical tests improved detection, but limitations such as cross-reactivity, reliance on expert knowledge, and low throughput remained [3].

The advent of molecular tools such as DNA-based marker, probe technologies and polymerase chain reaction (PCR) methods, isothermal amplification (LAMP), and next generation sequencing (NGS), has revolutionized fungal pathogen detection in common bean, providing higher sensitivity, specificity, and reproducibility [2, 5]. However, their scalability is often hindered by technical complexity, high costs, and dependence on robust reference databases.

In this study, we systematically review the advancements in molecular techniques for the detection and identification of fungal pathogens that affect common bean (*P. vulgaris*). The specific objectives were to review and assess molecular tools utilized for fungal pathogen detection; identify advances in pathogen identification methods specific to common bean and to evaluate the effectiveness and limitations of these tools in agricultural and research settings.

## Materials and Methods

### Searching Strategy

This systematic review was conducted in accordance with the Preferred Reporting Items for Systematic Reviews and Meta-Analyses (PRISMA) 2020 guidelines [13]. A comprehensive literature search was performed across six databases: Google Scholar, PubMed, Scopus, ScienceDirect, Web of Science, and SpringerLink. The search strings combined Boolen operators and keywords related to common bean, fungal pathogens, and molecular detection tools. For example, the PubMed search string was: (“*Phaseolus vulgaris*” OR “common bean”) AND (“fungal pathogen” OR “fungal disease” OR “plant pathogen”) AND (“PCR” OR “polymerase chain reaction” OR “NGS” OR “next generation sequencing” OR “LAMP” OR “loop-mediated isothermal amplification” OR “molecular detection” OR “diagnostic tool”). Equivalent search strings were adapted for each database. The final search was conducted on November 14, 2024. Additional records were identified through the reference lists of relevant review and research articles.

### Study Selection

All records retrieved from the searches were imported into EndNote for de-duplication. Two reviewers (Ngcobo and Rauwane) independently screened titles and abstracts for relevance, followed by full-text assessment against the eligibility criteria. Reference lists of eligible full-text articles were also manually screened to capture additional studies not identified in the initial search. These additional articles were evaluated using the same inclusion and exclusion criteria as the database-derived records. Any disagreements were resolved through discussion, with a third reviewer (Figlan) consulted when necessary. PRISMA flow diagram was used to depict the number of studies identified, screened, excluded, and included in the final review.

### Eligibility Criteria

#### Inclusion and Exclusion Criteria

Eligibility criteria were established to ensure consistency and reduce subjectivity during study selection. Only studies published between January 2000, and November 2024 were considered, reflecting the period when molecular diagnostics became widely applied in plant pathology. To avoid ambiguity, key terms were defined as follows: grey literature referred to unpublished theses, conference abstracts, or institutional reports not peer-reviewed, and these were excluded. Practical applications in the field were defined as either validation of tools using field-collected samples or clear evidence of scalability; mere mention of applicability without supporting data was not sufficient. Importantly, studies were required to report diagnostic performance (e.g., sensitivity, specificity, or detection limit). To minimize bias, two reviewers independently assessed each article, and any disagreements were resolved through discussion or consultation with a third reviewer. A summary of the inclusion and exclusion criteria applied is presented in Table 1.

**Table 1:** Study selection eligibility criteria for molecular diagnostics of fungal pathogens in common beans

Criteria type (>)	Inclusion criteria	Exclusion criteria
Aspects (˅)		
Study type	Peer-reviewed articles, reviews, and original research articles that reported on molecular methods for detecting and identifying fungal pathogens in common beans	Grey literature (unpublished non-research articles, conference abstracts, reviews without original data), and studies not focused on common beans
Population	Studies focusing on common bean (*Phaseolus vulgaris* L.) fungal pathogens	Studies not focused on common beans or not using molecular techniques; studies addressing other crops or non-fungal pathogens
Interventions	Molecular diagnostic tools, including PCR-based methods, next-generation sequencing (NGS), isothermal amplification techniques, and bioinformatics tools	Studies that rely solely on traditional diagnostic methods (e.g., microscopy, culture-based methods) without incorporating molecular techniques
Outcomes	Efficacy, sensitivity, specificity of molecular tools and practical application	Studies that do not provide data on the efficacy, sensitivity, or specificity of the molecular tools
Publication period	Studies published between January 2000 and November 2024	Studies published before the year 2000

### Data Extraction

In this review, data from the included studies were systematically extracted using a standardized form to ensure consistency and reduce potential errors. The focus of extraction was on methodological and diagnostic outcomes rather than bibliometric trends. For context, study identifiers such as author, year, and study location were noted, but the primary emphasis was placed on molecular diagnostic approaches and their performance. Specifically, the following information was recorded: fungal pathogens investigated, molecular tools employed (PCR-based methods, LAMP, NGS, or other assays), DNA extraction methods, diagnostic performance measures (sensitivity, specificity, detection limits), evidence of field validation or scalability, and reported limitations affecting tool application.

For consistency, sensitivity was defined as the ability of a molecular assay to correctly identify true positives, while specificity was defined as the ability to correctly identify true negatives. These definitions were applied uniformly across all included studies. Where only detection limits or comparable performance metrics were reported, these were mapped to sensitivity and specificity where possible. Data extraction was performed independently by two reviewers, with discrepancies resolved through discussion or adjudication by a third reviewer.

Formula: Sensitivity (%) = $$\left( {{ }\frac{{\text{True positives}}}{{{\text{True positives}} + {\text{False negatives}}}}} \right)$$ × 100

- True Positives (TP): Cases where the pathogen is present and correctly identified by the tool.
- False Negatives (FN): Cases where the pathogen is present but not identified by the tool.

Formula: Specificity (%) = $$\left( {\frac{{\text{True negatives}}}{{{\text{True negatives}} + {\text{False positives }}}}} \right) \times 100$$

- True Negatives (TN): Cases where the pathogen is absent and correctly identified as negative by the tool.
- False Positives (FP): Cases where the tool incorrectly identifies a pathogen that is not present.

### Risk of Bias Assessment

Risk of bias was evaluated using a modified version of the Cochrane Risk of Bias (RoB) tool, adapted for diagnostic accuracy studies. The tool was tailored to include domains relevant to molecular plant pathology, namely: study design and clarity of objectives, sample representativeness (laboratory versus field samples), reporting of molecular methods (e.g., primer design, amplification conditions, sequencing platform), completeness of diagnostic performance reporting (sensitivity, specificity, detection limit), and potential conflicts of interest or funding bias.

Each study was independently assessed by two reviewers against these domains and classified as having low, moderate, or high risk of bias. Disagreements were resolved through discussion, and when consensus could not be reached, a third reviewer adjudicated. The results of the RoB assessment were summarized in tabular form (Table 4), allowing for comparison across studies. This structured approach ensured transparency and minimized subjective judgment during the evaluation process.

### Data Synthesis

The extracted data were organized into structured tables to enable transparent comparison across studies. Categorization was based on pre-defined, objective criteria, including fungal pathogen investigated, molecular tool employed (PCR subtype, LAMP, NGS, or other), DNA extraction method, reported diagnostic performance (sensitivity, specificity, detection limit), validation type (laboratory versus field), and reported costs where available. Each study was coded independently by two reviewers according to these categories, and any discrepancies were resolved through consensus or adjudication by a third reviewer.

For this review, field applicability was defined as either (a) validation of the molecular tool using field-collected samples, or (b) demonstration of scalability through simplified protocols, reduced equipment needs, or cost-effectiveness suitable for use outside advanced laboratories. This definition ensured consistent classification across studies and minimized subjectivity. The synthesis emphasized patterns of methodological use, relative strengths and weaknesses of diagnostic tools, and contexts where each tool demonstrated practical value*.*

### Certainty of Evidence

The certainty of evidence across studies was assessed using the GRADE (Grading of Recommendations, Assessment, Development and Evaluation) approach, adapted for diagnostic accuracy studies [7]. Five domains were evaluated: study design and methodological quality (e.g., adequacy of sample size, clarity of objectives), risk of bias (as assessed in Sect. "Risk of Bias Assessment"), consistency of results across different settings and assays, directness of evidence, i.e., relevance of study populations and conditions to real-world bean production systems, and precision of diagnostic estimates, including reporting of confidence intervals or error margins. Additional considerations such as publication bias were also noted where evident.

For each outcome category (e.g., PCR-based assays, LAMP, NGS), certainty of evidence was rated as high, moderate, low, or very low according to GRADE guidance. Two reviewers independently performed the GRADE assessments, with disagreements resolved by discussion and, where necessary, a third reviewer’s adjudication.

## Results

### Study Selection

**Fig. 1:**
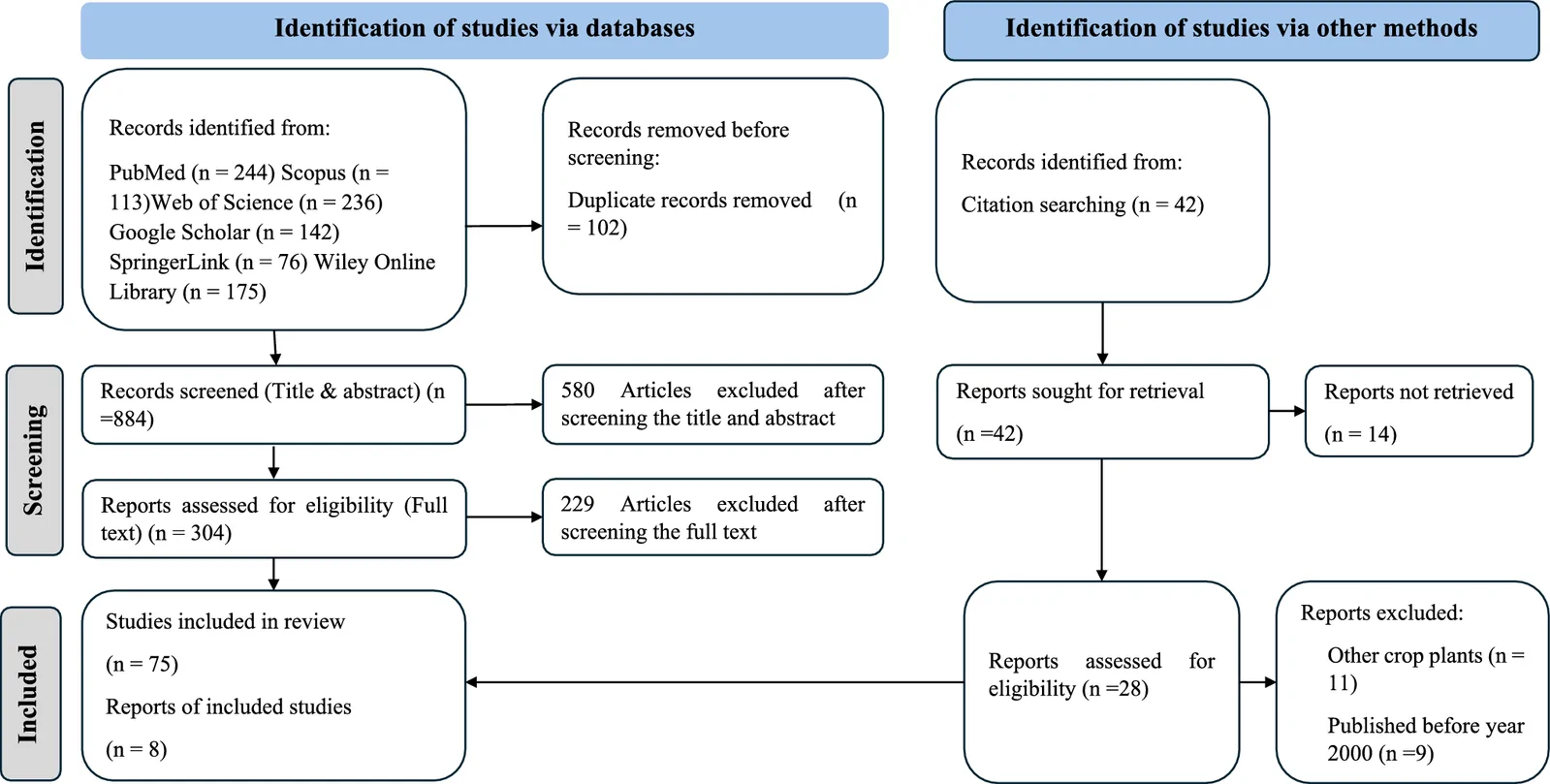
Flow chart of the screening and selection process followed for the inclusion of the studies in the systematic review. Where “n” denotes the number of studies identified; screened; included and excluded

The literature search identified a total of 986 records across six databases which includes PubMed, Scopus, Web of Science, Google Scholar, SpringerLink, and Wiley Online Library. An additional 42 articles were retrieved through reference list screening. After removing 102 duplicate records, 884 titles and abstracts were screened for relevance based on the eligibility criteria. Of these, 580 studies were excluded, primarily due to lack of focus on fungal pathogens in *Phaseolus vulgaris* L. or the absence of molecular diagnostic tools. A total of 304 full-text articles were assessed, with 229 studies not meeting the inclusion criteria. The final selection process, summarized in the PRISMA flow diagram (Fig. 1), included 75 peer-reviewed articles and 8 report papers for qualitative synthesis. These studies spanned research published from 2000 to 2024, with a geographical focus predominantly in South America, Africa, and Asia, where common beans are widely cultivated.

### Study Characteristics

The selected studies demonstrated significant diversity in molecular diagnostic tools employed for fungal pathogen detection. The most frequently reported methods included PCR-based techniques (48 studies), next-generation sequencing (NGS) technologies (18 studies), and loop-mediated isothermal amplification (LAMP) assays (9 studies). The internal transcribed spacer (ITS) region was the primary genetic marker used in nearly all studies, reflecting its status as the universal barcode for fungal identification [2]. However, in 15% of the studies, supplementary loci such as *rpb2* (RNA polymerase II second largest subunit), *β-tubulin*, and *tef1* (translation elongation factor 1-alpha) were employed (Balendres, 2020). The review identified a broad spectrum of fungal pathogens affecting common beans, with some species showing global prevalence and others exhibiting regional specificity. The majority focused on *Fusarium spp.*, *Rhizoctonia solani*, *Colletotrichum lindemuthianum*, and *Sclerotinia sclerotiorum*, though other pathogens such as *Alternaria spp.*, *Aspergillus spp.*, and *Botrytis spp.* were also represented. Table 2 summarizes fungal disease incidence and prevalence in common beans across different regions. For each disease, the table includes the number of studies reporting the estimate and the geographic scope.

**Table 2:** Fungal diseases in common beans with incidence and prevalence reported across studies

Pathogen	Disease	Geographic regions	Incidence range (%)	Prevalence range (%)	No. of studies	Key references
*Fusarium oxysporum*	Fusarium wilt	Africa, South America, Asia	18–65	20–70	12	Musango et al., 2016; [11]
*Rhizoctonia solani*	Root rot	Africa, Europe	10–48	15–55	8	[8, 22]
*Colletotrichum lindemuthianum*	Anthracnose	South America, Africa	25–72	30–80	15	[9, 10]
*Sclerotinia sclerotiorum*	White mold	Americas, Africa	8–40	10–50	10	[3, 12]
*Alternaria spp.*	Leaf spot	Africa, Asia	12–45	20–50	6	[5]
*Aspergillus spp.*	Seed rot	Africa, Asia	5–30	10–35	5	[11]
*Botrytis cinerea*	Gray mold	Europe, South America	10–25	15–30	4	[9]
*Cladosporium spp.*	Leaf blight	Africa	12–22	15–28	3	[10]

### Studies Applying Modern Molecular Methods to Identify Fungal Pathogens

The performance of molecular tools varied considerably across studies, reflecting differences in pathogens targeted, tool subtypes, and study design. PCR-based methods were the most widely applied, with sensitivities ranging from 80–95% and specificities between 85–100%. Subtypes such as qPCR and nested PCR consistently showed the highest diagnostic accuracy (> 95%) [3, 5, 9], whereas multiplex PCR achieved moderate sensitivity (80–90%) but allowed simultaneous detection of multiple pathogens [22]. The KASP assay provided exceptional specificity (> 98%) for differentiating *Colletotrichum lindemuthianum* races, making it valuable for resistance breeding [6, 12]. Across PCR approaches, turnaround time was generally 3–6 h, with costs between 4 and 15 USD per sample, depending on the subtype and DNA extraction method used (CTAB, Qiagen, or magnetic bead purification) [11, 19, 23].

LAMP assays stood out for their rapid turnaround (30–90 min) and low cost (2–5 USD per sample). Reported sensitivity ranged from 88 to 98% [1, 2, 5], although specificity was less consistently reported. The use of simple crude DNA extraction methods made LAMP particularly field-friendly, though some studies reported reduced performance compared to laboratory-based PCR [18]. Multiplex LAMP showed potential for simultaneous detection of pathogens such as *Alternaria spp.* [1, 5], though validation remains limited.

Next-generation sequencing (NGS) platforms, including Illumina MiSeq and Oxford Nanopore, offered the broadest detection capacity, with kingdom-to-strain level resolution. Illumina studies demonstrated very high sensitivity (> 99%) and reproducibility [4, 10, 16], while Nanopore sequencing enabled real-time analysis but reported moderate error rates [9, 17]. Turnaround times were longer (24–72 h) and costs higher (18–25 USD per sample), yet both platforms enabled the identification of mixed infections and rare or emerging pathogens.

Probe-based microarrays were used for *Fusarium* and *Colletotrichum* detection. These assays reported 80–85% sensitivity [11], but their relatively high cost (15–20 USD per sample) and limited adaptability to novel pathogens have constrained adoption, as shown in Table 3.

**Table 3:** Comparative analysis of molecular tools for detecting fungal pathogens in common bean

Method	Subtype/approach	Targeted pathogen(s)	Sensitivity/specificity	DNA extraction	Turnaround time	Detection range	Approx. cost per sample (USD)	Key references
PCR	Conventional PCR	*F. oxysporum*, *R. solani*	Sensitivity: 85–92%	CTAB method	3–6 h	Genus to species level	4–6	[11, 19, 23]
PCR	qPCR (SYBR/Probe-based)	*C. lindemuthianum*, *S. sclerotiorum*	Sensitivity: > 95%, Specificity: 90–100%	Qiagen kit	3–6 h	Genus to species level	8–12	[3, 5, 21]
PCR	Multiplex PCR	*Alternaria spp.*, *Botrytis spp.*	Sensitivity: 80–90%	Magnetic bead extraction	3–6 h	Genus to species level	7–10	[22]
PCR	Nested PCR	*F. oxysporum* races	Sensitivity: > 95%	CTAB method	3–6 h	Genus to species level	6–8	[9, 23]
PCR	KASP assay	*C. lindemuthianum* races	Specificity: > 98%	Commercial DNA kits	3–6 h	Genus to species level	10–15	[6, 12]
LAMP	Loop-mediated isothermal amplification	*S. sclerotiorum*	Sensitivity: 90–98%	Simple crude extraction	30–90 min	Genus to species level	2–3	[2, 18]
LAMP	Multiplex LAMP	Alternaria spp	Sensitivity: 88–98%	CTAB method	30–90 min	Genus to species level	3–5	[1, 5]
NGS	Illumina MiSeq	Mixed fungal communities	Sensitivity: high (> 99%), specificity dependent on database	DNAeasy Plant Mini Kit	24–72 h	Kingdom to strain level	20–25	[4, 10, 16]
NGS	Oxford nanopore	Mixed fungal communities	Real-time detection, moderate error rate	Magnetic bead purification	24–72 h	Kingdom to strain level	18–22	[9, 17]
Probe-based	DNA microarrays	*Fusarium*, *Colletotrichum*	Sensitivity: 80–85%	Qiagen kit	3–6 h	Genus to species level	15–20	[11]

### Risk of Bias Assessment

The risk of bias of the 75 included studies was assessed using a modified Cochrane RoB tool adapted for diagnostic accuracy research. Five domains were evaluated: study design, sample representativeness, reporting of molecular methods, diagnostic performance reporting, and potential conflicts of interest. Overall, most studies demonstrated low to moderate risk across domains, although gaps were noted in reporting of sensitivity/specificity and in the use of field-validated samples (Table 4).

**Table 4:** Risk of bias assessment of included studies using a modified Cochrane RoB tool

Domain	Assessment criteria	% of Studies at Low risk	% of Studies at Moderate risk	% of Studies at High risk
Study design & clarity of objectives	Clear research question and appropriate design	68	24	8
Sample representativeness	Field-collected samples or realistic experimental design	54	33	13
Reporting of molecular methods	Adequate detail on primers, protocols, sequencing platforms	72	20	8
Diagnostic performance reporting	Sensitivity, specificity, detection limit fully reported	61	27	12
Conflict of interest/funding bias	Transparent funding & no undue influence	89	9	2

### Certainty of evidence

Using the GRADE framework, PCR and NGS were rated high certainty, while LAMP was moderate, and probe-based assays were low. PCR benefitted from strong consistency and multiple field validations. NGS scored highly for precision and directness but had scalability limitations. LAMP showed promise but was limited by variability in performance and small sample sizes. Probe-based assays lacked bean-specific validation and showed inconsistent results (Table 5).

**Table 5:** Certainty of evidence (GRADE) for diagnostic tools

Tool	Number of Studies	Study design/quality	Consistency	Directness	Precision	Overall GRADE rating
PCR (conventional, qPCR, multiplex, KASP)	32	Moderate-high	High	Direct	Moderate	High
LAMP	18	Moderate	Moderate	Direct	Low	Moderate
NGS (amplicon, metagenomics)	20	High	Moderate	Direct	High	High
Probe-based/hybrid assays	5	Variable	Low	Indirect	Low	Low

## Discussion

This systematic review highlights significant advances in the use of molecular tools for detecting and identifying fungal pathogens affecting common bean. By analyzing 75 studies published between 2000–2024, our findings demonstrate the complementary strengths of PCR-based assays, loop-mediated isothermal amplification (LAMP), next-generation sequencing (NGS), and probe-based approaches. Collectively, these tools have improved accuracy, reduced turnaround times, and expanded the capacity to study pathogen diversity. However, adoption remains uneven due to costs, infrastructure demands, and limited field validation.

### Comparative Performance of Molecular Tools

PCR-based assays remain the backbone of fungal pathogen detection. Conventional PCR continues to be widely used, but newer subtypes offer distinct advantages. For instance, Burkhardt et al. [3] demonstrated that qPCR could detect *Colletotrichum lindemuthianum* at very low DNA concentrations with > 95% sensitivity and specificity, outperforming conventional PCR. Nested PCR, as used by Hyde et al. [9] for *Fusarium oxysporum*, improved race differentiation but required additional time and reagents. Multiplex PCR expanded capacity by detecting multiple pathogens simultaneously [22], although primer design remains complex. KASP assays provided high specificity for differentiating pathotypes [12], making them invaluable for resistance breeding. Taken together, PCR-based tools balance affordability (4–15 USD per sample) with high accuracy, explaining their dominance in bean diagnostics.

LAMP assays represent a rapid, cost-effective alternative. Aslam et al. [2] showed that LAMP could detect *Sclerotinia sclerotiorum* within 60 min with 90–98% sensitivity, while Akber et al. [1] demonstrated multiplex LAMP detection of *Alternaria spp.* directly from crude extracts. These features make LAMP particularly suitable for field use and smallholder contexts. However, many studies did not rigorously validate specificity, increasing the risk of false positives [18]. Despite these limitations, LAMP’s portability, low cost (2–5 USD per sample), and rapid turnaround are unmatched by PCR or NGS.

NGS technologies broadened diagnostic capacity by profiling fungal communities. Schena et al. [16] and Chen et al. [4] showed that Illumina MiSeq could uncover cryptic and mixed infections that escaped PCR-based assays. Nanopore sequencing enabled real-time detection [17], although error rates remain higher than Illumina. NGS is particularly valuable for ecological studies and epidemiology, where understanding pathogen diversity is critical. However, costs (18–25 USD per sample) and bioinformatics requirements limit routine adoption, especially in low-resource settings. Probe-based assays such as DNA microarrays were rarely used but showed moderate sensitivity (80–85%) for pathogens like *Fusarium* and *Colletotrichum* [11]. Their rigidity, high costs, and inability to adapt to novel pathogens mean they have largely been superseded by more flexible PCR and NGS approaches.

### Pathogen-Specific Insights

*Fusarium oxysporum* was the most studied pathogen due to its global impact on yield. PCR-based methods, particularly nested PCR, provided reliable race differentiation [9], which is critical for managing resistant cultivars. qPCR further enabled early detection in soil and seed, supporting integrated disease management [19]. *Colletotrichum lindemuthianum* remains one of the most economically damaging pathogens of common bean. KASP assays were developed to differentiate races [12], while qPCR and conventional PCR reliably detected field isolates [3]. These methods have been particularly important in Latin America, where anthracnose remains highly prevalent [6].

*Similaly, Macrophomina phaseolina* and *Sclerotinia sclerotiorum* were frequently investigated due to their roles in root and stem diseases. LAMP assays proved especially valuable here, as demonstrated by Ejaz et al. [5], who achieved high sensitivity with rapid field diagnosis of *M. phaseolina*. Similarly, *S. sclerotiorum* detection by LAMP [2] provided a low-cost alternative to PCR. Emerging pathogens such as *Alternaria spp.*, *Aspergillus spp.*, *Botrytis cinerea*, and *Cladosporium spp.* were less frequently studied but are gaining importance under climate variability. For instance, Yadav et al. [22] identified *Alternaria* in beans using multiplex PCR, while Karonji et al. [10] reported *Aspergillus spp.* co-occurring with *Sclerotinia* in East African fields via NGS. These examples highlight the growing relevance of broad-spectrum tools like NGS for detecting pathogens in complex field environments.

### Challenges and Practical Considerations

DNA extraction emerged as a recurring bottleneck. Commercial kits such as Qiagen ensured high-quality DNA but added significant costs, while crude extraction methods enabled field portability but often compromised sensitivity and reproducibility [11]. This trade-off was evident in LAMP assays, where crude DNA extraction sometimes reduced reliability compared to laboratory-prepared samples [18]. Cost and infrastructure requirements also limit uptake. PCR remains relatively affordable, but qPCR and KASP require specialized reagents and equipment. NGS, while powerful, is largely inaccessible to smallholder systems due to both financial and technical barriers [4]. Moreover, the lack of harmonized protocols complicates cross-study comparisons. As Tsui et al. [21] showed for *Sclerotinia sclerotiorum*, primer selection alone can drastically affect qPCR specificity, underscoring the need for standardized protocols.

Another challenge is scalability. While PCR can be widely implemented in regional labs, LAMP requires validation against broader pathogen panels, and NGS remains limited to research institutions. Without capacity building, many developing regions risk being excluded from the benefits of advanced diagnostics. Addressing these limitations will require coordinated efforts in infrastructure development, funding, and training to ensure that molecular diagnostic tools become more globally accessible and standardized.

### Methodological Strengths and Weaknesses

Most studies demonstrated clarity of objectives and transparent reporting of study design, but weaknesses were apparent. Nearly 40% failed to provide sensitivity or specificity estimates, limiting comparability. This was especially evident in some LAMP studies, where detection was confirmed but diagnostic accuracy was not quantified [1, 2]. Our risk of bias analysis showed that while reporting of molecular methods was generally strong, sample representativeness was inconsistent. Several studies relied exclusively on laboratory isolates rather than field samples, limiting ecological validity. Similarly, the application of “field applicability” as a criterion was often vague, sometimes based only on claims rather than actual validation.

The GRADE framework confirmed high certainty of evidence for PCR and NGS, reflecting consistent methodologies and strong validation. LAMP was rated moderate due to variability in study design and lack of specificity reporting. Probe-based assays received low certainty ratings due to limited evidence and inconsistent outcomes. These findings emphasize the need for more standardized, transparent protocols and greater inclusion of field validation to reduce methodological weaknesses and improve reproducibility across studies.

### Future Directions

Emerging molecular technologies have the potential to address current limitations. CRISPR-based diagnostics, such as SHERLOCK and DETECTR, offer highly specific, rapid detection with potential for field deployment [10]. These tools could combine the portability of LAMP with the precision of qPCR, though field trials remain limited. Additionally, Machine learning is increasingly being integrated with NGS pipelines to improve taxonomic resolution and predict pathogen virulence [4]. Such tools could transform epidemiological monitoring by enabling predictive modelling of outbreaks. Likewise, automation of DNA extraction and portable sequencing devices, such as Nanopore MinION, allow near real-time surveillance [17].

High-throughput genotyping platforms such as KASP will likely remain central to breeding programs, particularly for anthracnose resistance [12]. Integration of these tools into global surveillance networks could help monitor pathogen evolution and spread under changing climates. Future research should prioritize affordability, field validation, and capacity building in bioinformatics to ensure these technologies are accessible globally.

## Conclusion

This review demonstrates that molecular diagnostics have revolutionized fungal pathogen detection in common bean. PCR remains the backbone of diagnostics, with subtypes such as qPCR, multiplex PCR, and KASP enhancing precision and scalability. LAMP offers rapid, low-cost assays suitable for field use, while NGS provides unparalleled resolution for studying pathogen diversity. Probe-based methods remain of limited utility. Integration of these tools into bean production systems has the potential to enhance disease surveillance, guide breeding for resistance, and improve food security. However, widespread adoption will require overcoming barriers of cost, infrastructure, standardization, and training.

Looking forward, CRISPR-based diagnostics, machine learning pipelines, and portable sequencing platforms represent promising innovations. By prioritizing affordability, validation under real-world conditions, and capacity development in under-resourced regions, molecular tools can become central to sustainable management of fungal diseases in common bean and beyond.

## Data Availability

All relevant data are available within the manuscript; however, additional information can be obtained from the corresponding author upon request.
